# Very Early Severe Posttransplant Recurrent Antineutrophil Cytoplasmic Antibody-Associated Glomerulonephritis after Kidney Transplantation: Two Case Reports

**DOI:** 10.1155/2022/9740225

**Published:** 2022-03-03

**Authors:** Reda Laamech, Hamza Naciri-Bennani, Diane Giovannini, Johan Noble, Bénédicte Janbon, Paolo Malvezzi, Thomas Jouve, Lionel Rostaing

**Affiliations:** ^1^Nephrology, Hemodialysis, Apheresis and Kidney Transplantation Department, Grenoble University Hospital, Grenoble, France; ^2^Grenoble Alpes University, Grenoble, France; ^3^Laboratory of Pathology, Grenoble University Hospital, Grenoble, France

## Abstract

Successful kidney transplantation (KTx) in patients with antineutrophil cytoplasmic antibody-associated vasculitis (AAV) has been reported with excellent patient and graft survival rates. The recurrence of AAV in transplant recipients is rare, and its mechanisms of action are not clearly known. The optimum time for KTx and the relevance of ANCA titer at the time of transplantation remain controversial. We report two cases of extremely rapid recurrent AAV after renal transplantation; both were still ANCA-positive at the time of transplantation, which led us to question the pathogenesis of ANCA antibodies in recurrence in a kidney allograft. Apheresis plus immunosuppressive therapies were ineffective in the first case and the patient became dialysis-dependent, whereas in the second case methylprednisone pulses plus rituximab infusions resulted in long-lasting remission.

## 1. Introduction

End-stage kidney disease (ESKD) associated with antineutrophil cytoplasmic autoantibody-associated vasculitis (AAV) is considered an indication for renal transplantation. It improves the quality of life and life expectancy in patients with ESKD. Indeed, kidney transplantation (KTx) has been successfully performed in AAV patients [1, 2], and several studies confirm the survival benefits of renal transplantation compared to maintenance dialysis [3]. Nonetheless, AAV relapses have been reported many times; in pooled analyses from multiple studies, between 5 and 6% of transplant recipients suffer a relapse [4], which often affects allograft outcomes. These cases display different mechanisms and risk factors, such as the timing of renal transplantation after AAV diagnosis, the role of immunosuppressive maintenance in patients during chronic dialysis period, and antineutrophil cytoplasmic antibody (ANCA) titers at the time of transplantation. However, a link between ANCA titers and allograft failure has not been clearly established, although there is a trend showing a link between ANCA titers at the time of transplantation and the risk of relapse and overall graft survival [5].

Direct and indirect involvement of ANCA antibodies in AAV disease has been widely discussed. Herein, we report two cases of patients presenting with AAV and having high ANCA titers at the time of transplantation; they also had rapid AAV recurrence on the allograft kidney with a primary failure in one case.

## 2. Case Report/Case Presentation

The case presentations were conducted ethically in accordance with the World Medical Association Declaration of Helsinki.

### 2.1. Case 1

A 54-year-old man was admitted for a kidney transplant. He had been on hemodialysis for 21 months because of ANCA-associated ESKD; he was almost anuric. When he was diagnosed, he was treated with pulses of methylprednisolone and two IV injections (one month apart) of cyclophosphamide 0.6 g/m^2^, but there was no improvement. No maintenance immunosuppressive treatment was introduced. He received a living-related ABO and HLA-compatible KTx on July 9, 2020. He was fully matched for class II HLA antigens and was not HLA sensitized at pretransplant. He received induction therapy with antithymocyte globulins (ATG), in addition to tacrolimus, mycophenolate mofetil (MMF), and steroids, i.e., methylprednisone 500 mg preoperatively and then 500 mg on days 1 and 2. He recovered immediate diuresis and serum creatinine began to decrease 12 hours after surgery (from 6.9 to 5.4 mg/dL). However, urine output suddenly decreased on day 1 after transplantation. Serum creatinine (sCr) then rose from 5.4 to 6.9 mg/dL.

On day 1, posttransplant serum ANCA titer was >1280 UI/mL with an MPO specificity of >740 UI/mL. No ANCA serum titer was performed immediately before kidney transplantation.

**Figure 1:**
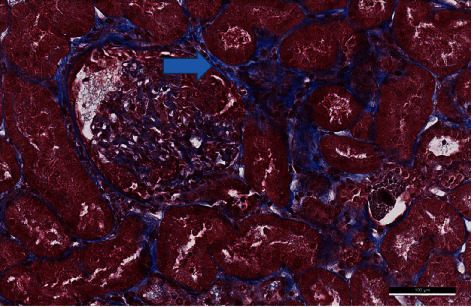
One glomerulus highlighted a cellular crescent—blue arrow (blue trichrome, high power field).

**Figure 2:**
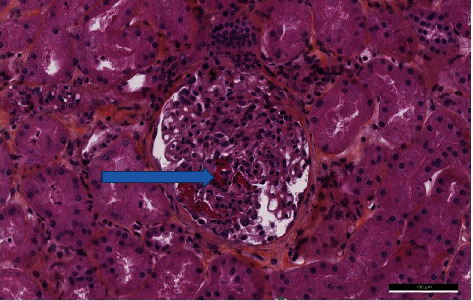
Another glomerulus highlighted a fibrinoid necrosis—blue arrow (PAS staining, high power field).

On day 2, Doppler ultrasound evaluation of the kidney allograft was normal. Surgery was performed in search of a vascular plication, but no explanation for allograft failure was found. A biopsy realized on day 7 after transplantation revealed necrotizing vasculitis with fibrinoid necrosis and extracapillary proliferation, confirming AAV relapse (shown in Figures 1 and 2). Proteinuria was dosed at 1.7 g/L; there was no associated hematuria. We implemented plasmapheresis sessions (nine over a 14-day period), plus three methylprednisolone pulses (10 mg/kg each) and rituximab (375 mg/m^2^) on postop days 9, 17, 24, and 37. This resulted in a sharp decrease in the anti-MPO titer (from >740 to 80 U/mL). However, the patient remained dialysis-dependent.

**Figure 3:**
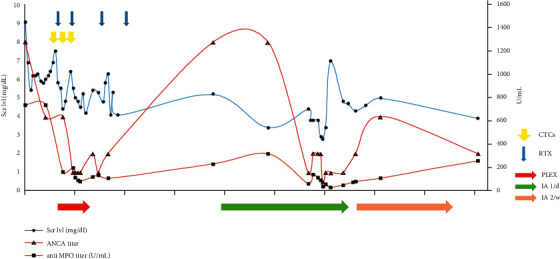
Case 1: serum creatinine level, ANCA titer, and anti-MPO level evolution over time. Scr lvl, serum creatinine level; CTCs, methylprednisone pulse; RTX, rituximab; PLEX, plasmapheresis; IA 1/d, immunoadsorption one per day; IA 2/w, immunoadsorption, two per week; ANCA, antineutrophil cytoplasmic antibody; MPO, myeloperoxidase.

Allograft biopsies on postop days 15 and 21 were scored according to Banff classification as i1, t1, g1 ptc2, and C4d0 and revealed persisting fibrinoid necrosis and extracapillary proliferation, with no histological improvement (D21 vs. previous biopsies). On postop day 60, we observed a rebound in ANCA titer to >1280 UI/mL and in anti-MPO titer of 317.8 U/mL with a patient still dialysis-dependent. We therefore decided to implement seven semispecific immunoadsorption (IA) using a Globaffin® immunoadsorber. Maintenance immunosuppression was based on MMF 500 mg bid, prednisone 20 mg/d, and tacrolimus in order to achieve trough levels between 6 and 8 ng/mL. A follow-up kidney biopsy was performed at 3 months postop showing no improvement in extracapillary proliferation or fibrinoid necrosis, but there were no signs of allograft rejection. We then decided to perform two IA sessions for 2 weeks. However, unfortunately, the patient remained dialysis-dependent with predialytic serum creatinine remaining at >5 mg/dL, even though ANCAs with MPO specificity remained low at 55–70 UI/mL. The relevant biological markers for evolution are shown in Figure 3.

One month later, i.e., postop day 120, there was no improvement in kidney function. We decided that further therapeutic approaches were not possible. The patient remained dialysis-dependent; MMF was stopped, tacrolimus (trough levels 5–6 ng/mL) and prednisone (10 mg/d) were maintained; unfortunately, he died of septic shock at 6 months after transplantation. His brother (the kidney donor) has given written informed consent to publish the case.

### 2.2. Case 2

An 84-year-old man with chronic kidney disease of unknown origin had an episode of acute kidney injury. The estimated glomerular filtration rate (eGFR) declined from 30 to 10 ml/min/1.73 m^2^ in one month. No kidney biopsy was performed. He went onto maintenance dialysis in May 2018. The first assessments showed high levels of anti-MPO titers, at 640 UI/L. The first kidney biopsy (January 4, 2018) showed diffuse thickening of the glomerular basement membrane (GBM) throughout all the glomeruli without hypercellularity. Immunofluorescence microscopy revealed a diffuse granular pattern of immunoglobulin G without C3 staining along the GBM. PLA2R antibodies were negative. There were no signs of AAV on the kidney biopsy; thus, membranous nephropathy was diagnosed. He received no immunosuppressive therapy for that condition.

**Figure 4:**
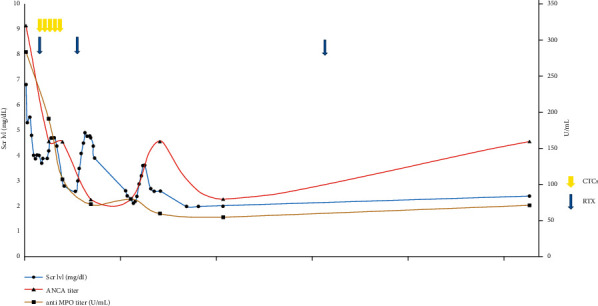
Case 2: serum creatinine level, ANCA titer, and anti-MPO level evolution over time. Scr lvl, serum creatinine level; CTCs, methylprednisone pulse; RTX, rituximab; ANCA, antineutrophil cytoplasmic antibody; MPO, myeloperoxidase.

**Figure 5:**
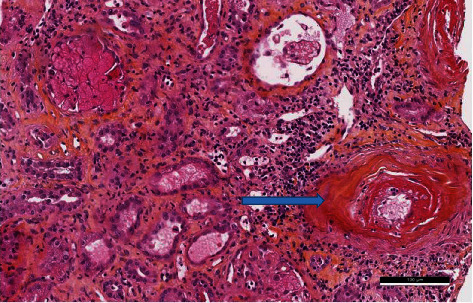
HES high power field: an apoplectic glomerulus and two medium-sized arteries presenting with fibrinoid necrosis (blue arrow).

He received a transplant on October 24, 2019, from a deceased donor. He had no anti-HLA antibodies at pretransplant. He was given induction therapy with ATG, along with tacrolimus, everolimus, and prednisone. The patient initially had a good urine output of >1 L/day and serum creatinine decreased to 3.7 mg/dL on postoperative day 8. At 2 weeks, serum creatinine stabilized at 3.95 mg/dL. An allograft biopsy, realized on postop day 11, revealed necrotizing vasculitis compatible with relapsing AAV and/or cellular rejection grade 3. Histological injuries included glomerular and arteriolar thrombotic microangiopathy, acute tubular necrosis, g1, cg2, ptc1, C4d0, t1 + i0, FIAT1, v0, cv3, ah3, and aah0 (shown in Figure 4). Biology revealed ANCA positivity with MPO specificity reaching 285 UI/mL at the time of kidney transplantation. Treatment included pulses of methylprednisone (500 mg daily dose for 5 days), two injections of rituximab (375 mg/m^2^ on postop days 12 and 26): it provided good efficacy, i.e., serum creatinine was reduced to 2.8 mg/dL and ANCA titer was decreased to 80 UI/mL. The relevant biological markers for evolution are shown in Figure 5.

Maintenance therapy included perfusions of rituximab (375 mg/m^2^ every 6 months). Allograft function was stabilized with eGFR at 20 mL/min/1.73 m^2^. At postop 9 months, serum creatinine level was 2.38 mg/dL and proteinuria was 0.5 g/L, with no anti-HLA alloantibodies. Anti-MPO was 70.7 UI/L and a biopsy showed tubulointerstitial inflammation without any signs of AAV. By the end of 2020 (postop month 14), the patient was admitted into the hospital for SARS-CoV-2 infection and died of SARS-CoV-2-related pneumopathy. His late wife has given written informed consent to publish the case.

## 3. Discussion

In pooled analyses from multiple studies, 5.4% of AAV patients with a transplant have a relapse. The relapse rate after transplantation seems to have declined over the last few years, with rates of 0.01–0.10 per patient/year reported in studies older than 10 years compared to rates of 0.01–0.03 per patient/year in more recent studies. Although the frequency of AAV recurrence after kidney transplantation is rare, there is a one-in-three risk of graft loss within 5 years [6].

The timing of KTx in patients with AAV is still being discussed. Little et al. [5] found that the strongest predictor for graft loss and mortality in AAV patients was when transplantation was conducted shortly (<1 year) after the induction of a remission. ANCA positivity per se, at the time of transplantation, was not associated with adverse graft survival. The finding of severe vascular lesions on a transplant biopsy was the only factor significantly associated with graft failure. However, the impact of ANCA is not definitive from the study of Little et al., as graft failure could have been induced by several other factors.

Gera et al. [7] in their single-center experience reported that AAV needs to be in clinical remission at the time of transplantation. Both the 2012 Kidney Disease Improving Global Outcomes Guidelines for Glomerulonephritis (KDIGO) and the 2005 Canadian Society of Transplantation Consensus Guidelines recommend a period of 1 year of clinical remission prior to transplantation.

The necessity of ANCA testing before proceeding with KTx is less fully agreed upon. ANCA positivity alone is not a contraindication for transplantation. However, Gera et al. [7] reported a trend towards a higher relapse rate among patients that had a positive ANCA test just before KTx; that is, 13% vs. 5% in those having no longer detectable ANCA at the time of transplantation.

Nonetheless, rapid relapse and allograft failure are rarely reported in the literature. The time to relapse is highly variable, ranging from weeks to more than 10 years, with a mean time of 26.5 months posttransplant [8]. For example, the average time to relapse was 30.9 months (range: 4–84 months) in the pooled analysis performed by Nachman et al. [9]. Lobbedez et al. [10] reported a recurrence of ANCA-positive glomerulonephritis immediately after renal transplantation. The renal biopsy (performed on postoperative day 6) showed crescentic necrotizing glomerulonephritis and the p-ANCA titer was positive at 1 : 400 on the day of renal transplantation. This relapse was successfully treated with cyclophosphamide, intravenous immunoglobulins (IVIg), steroid pulses, cyclosporine, and plasmapheresis sessions. The ANCA titer became negative by the end of the treatment.

Kai Ming Chow et al. [11] reported a case of rapid (1 month post-KTx) recurrence of ANCA-negative glomerulonephritis after KTx. Five sessions of plasmapheresis with cyclophosphamide did not improve renal function, and the patient remained dialysis-dependent.

These findings along with ours led us to question the pathogenesis of ANCA antibodies. Several animal models of AAV have been established [12]. This shows that ANCAs not only serve as a biomarker for AAV but also have pathogenic potential in themselves. But ANCA titers do not necessarily reflect disease activity. The lack of a correlation between ANCA titers and the activity of vasculitis disease could be explained by differences in epitopes and the affinity of ANCAs [12].

Also, ANCAs cannot be detected in some patients with clinical manifestations of MPO-AAV; laboratory tests can give false-negative results because fragments of ceruloplasmin can bind to the ANCAs in the serum. The absence of ANCAs in necrotizing lesions can be explained by the presence of neutrophil extracellular traps (NETs), which induce digestion of immunoglobulins by neutrophil elastase derived from NET-forming neutrophils. All these elements make the underlying mechanisms hard to elucidate and demonstrate the pathogenesis of the ANCA complex [13].

Such rapid recurrences among patients receiving a kidney allograft, with positive ANCA titers at the time of transplantation, have led us to discuss the pathogenesis of ANCA and the importance of finding a biomarker for disease activity (still not yet available). This could inform us of the pertinence and safety of renal transplantation in AAV patients, as well as the prognosis of AAV in kidney-transplant recipients.

## 4. Conclusion

We report two cases of AAV in recipients of allograft kidneys. Both cases presented with very early posttransplant allograft dysfunction in the setting of a high titer level of ANCAs and MPO specificity at the time of kidney transplantation. Pathogenicity of ANCAs has been a subject of controversy as no evidence has been provided since its discovery in the 1980s of cases where it has occurred on native kidneys, allograft relapses, or in de novo post-KTx, where immunosuppressive therapy is thought to avoid outcomes. Further exploration of the mechanisms behind this phenomenon should be made.

## Data Availability

Data are available upon request.

## References

[1] Sagmeister M. S., Grigorescu M., Schönermarck U. (2019). Kidney transplantation in ANCA-associated vasculitis. *Journal of Nephrology*.

[2] Schmitt W. H., Van Der Woude F. J. (2003). Organ transplantation in the vasculitides. *Current Opinion in Rheumatology*.

[3] Wolfe R. A., Ashby V. B., Milford E. L. (1999). Comparison of mortality in all patients on dialysis, patients on dialysis awaiting transplantation, and recipients of a first cadaveric transplant. *New England Journal of Medicine*.

[4] Briganti E. M., Russ G. R., McNeil J. J., Atkins R. C., Chadban S. J. (2002). Risk of renal allograft loss from recurrent glomerulonephritis. *New England Journal of Medicine*.

[5] Little M. A., Hassan B., Jacques S. (2009). Renal transplantation in systemic vasculitis: when is it safe?. *Nephrology Dialysis Transplantation*.

[6] Göçeroğlu A., Rahmattulla C., Berden A. E., Reinders M. E. J., Wolterbeek R., Steenbergen E. J. (2016). The Dutch transplantation in vasculitis (DUTRAVAS) study: outcome of renal transplantation in antineutrophil cytoplasmic antibody-associated glomerulonephritis. *Transplantation*.

[7] Gera M., Griffin M.-D., Specks U., Leung N., Stegall M.-D., Fervenza F.-C. (2007). Recurrence of ANCA-associated vasculitis following renal transplantation in the modern era of immunosupression. *Kidney International*.

[8] Marco H., Mirapeix E., Arcos E. (2013). Long-term outcome of antineutrophil cytoplasmic antibody-associated small vessel vasculitis after renal transplantation. *Clinical Transplantation*.

[9] Nachman P. H., Segelmark M., Westman K. (1999). Recurrent ANCA-associated small vessel vasculitis after transplantation: a pooled analysis. *Kidney International*.

[10] Lobbedez T., Comoz F., Renaudineau E., Pujo M., Ryckelynck J. P., Hurault de Ligny B. (2003). Recurrence of ANCA-positive glomerulonephritis immediately after renal transplantation. *American Journal of Kidney Diseases*.

[11] Chow K. M., Wang A. Y., Mac-Moune Lai F., Wong T. Y., Li P. K. (2001). Rapid recurrence of ANCA-negative pauci-immune vasculitis after cadaveric renal transplantation. *American Journal of Kidney Diseases*.

[12] Nakazawa D., Masuda S., Tomaru U., Ishizu A. (2019). Pathogenesis and therapeutic interventions for ANCA-associated vasculitis. *Nature Reviews Rheumatology*.

[13] Roth A. J., Ooi J. D., Hess J. J. (2013). Epitope specificity determines pathogenicity and detectability in ANCA-associated vasculitis. *Journal of Clinical Investigation*.

